# Is There a Link between Obesity Indices and Skin Autofluorescence? A Response from the ILERVAS Project

**DOI:** 10.3390/nu15010203

**Published:** 2022-12-31

**Authors:** Enric Sánchez, Marta Sánchez, Carolina López-Cano, Marcelino Bermúdez-López, José Manuel Valdivielso, Cristina Farràs-Sallés, Reinald Pamplona, Gerard Torres, Dídac Mauricio, Eva Castro, Elvira Fernández, Albert Lecube

**Affiliations:** 1 Endocrinology and Nutrition Department, University Hospital Arnau de Vilanova, Obesity, Diabetes and Metabolism (ODIM) Research Group, IRBLleida, University of Lleida, 25198 Lleida, Spain; 2 Vascular and Renal Translational Research Group, IRBLleida, Red de Investigación Renal, Instituto de Salud Carlos III (RedinRen-ISCIII), 25198 Lleida, Spain; 3 Centre d’Atenció Primària Cappont, Gerència Territorial de Lleida, Institut Català de la Salut, Research Support Unit Lleida, Fundació Institut Universitari per a la Recerca a l’Atenció Primària de Salut Jordi Gol i Gorina (IDIAPJGol), 08007 Barcelona, Spain; 4 Department of Experimental Medicine, IRBLleida, University of Lleida, 25003 Lleida, Spain; 5 Respiratory Medicine Department, University Hospital Arnau de Vilanova and Santa María, Group of Translational Research in Respiratory Medicine, IRBLleida, University of Lleida, 25198 Lleida, Spain; 6 Centro de Investigación Biomédica en Red de Enfermedades Respiratorias (CIBERES), Instituto de Salud Carlos III (ISCIII), 28029 Madrid, Spain; 7 Department of Endocrinology and Nutrition, Hospital de la Santa Creu i Sant Pau, Sant Pau Biomedical Research Institute (IIB Sant Pau), 08025 Barcelona, Spain; 8 Centro de Investigación Biomédica en Red de Diabetes y Enfermedades Metabólicas Asociadas (CIBERDEM), Instituto de Salud Carlos III (ISCIII), 28029 Madrid, Spain

**Keywords:** adipose tissue, advanced glycation end-products, body composition, cardiometabolic risk, cardiovascular risk factors, novel targets, obesity, skin autofluorescence

## Abstract

There is controversial information about the accumulation of advanced glycation end-products (AGEs) in obesity. We assessed the impact of total and abdominal adiposity on AGE levels via a cross-sectional investigation with 4254 middle-aged subjects from the ILERVAS project. Skin autofluorescence (SAF), a non-invasive assessment of subcutaneous AGEs, was measured. Total adiposity indices (BMI and Clínica Universidad de Navarra-Body Adiposity Estimator (CUN-BAE)) and abdominal adiposity (waist circumference and body roundness index (BRI)) were assessed. Lean mass was estimated using the Hume index. The area under the receiver operating characteristic (ROC) curve was evaluated for each index. Different cardiovascular risk factors (smoking, prediabetes, hypertension and dyslipidemia) were evaluated. In the study population, 26.2% showed elevated SAF values. No differences in total body fat, visceral adiposity and lean body mass were detected between patients with normal and high SAF values. SAF levels showed a very slight but positive correlation with total body fat percentage (estimated by the CUN-BAE formula) and abdominal adiposity (estimated by the BRI). However, none of them had sufficient power to identify patients with high SAF levels (area under the ROC curve <0.52 in all cases). Finally, a progressive increase in SAF levels was observed in parallel with cardiovascular risk factors in the entire population and when patients with normal weight, overweight and obesity were evaluated separately. In conclusion, total obesity and visceral adiposity are not associated with a greater deposit of AGE. The elevation of AGE in obesity is related to the presence of cardiometabolic risk.

## 1. Introduction

Obesity is a multifactorial chronic disease that can shorten the quality of life and life expectancy of patients due to its high morbidity and mortality [1]. While this is clearly established, population studies have revealed that more than 30% of patients with obesity do not present associated metabolic pathology, which has given rise to the concept of “metabolically healthy obesity” [2]. However, these patients have shown a higher risk of both diabetes and cardiometabolic disease in the medium–long term. It has been hypothesized that this is a possible initial phase prior to the development of comorbidities [3,4,5].

To date, the trigger for the development of these comorbidities is unknown. However, among the different hypotheses, the possible role of inflammatory adaptation against tissue hypoxia produced by the expansion of white adipose tissue is becoming increasingly relevant [6,7,8]. This continuous hypoxia facilitates a change towards a proinflammatory profile that enhances the secretion of cytokines such as tumor necrosis factor alpha, interleukin-6 or hypoxia inducible factor type 1 with the consequent increase in acute phase indicators such as C-reactive protein and fibrinogen [6,9,10]. These factors are related to the appearance of both local and systemic insulin resistance, endothelial dysfunction and arteriosclerosis, as well as a higher rate of cardiovascular events [11,12].

Hypoxia, along with a proinflammatory pattern and oxidative stress, are common features of obesity, all of which have been associated with increased protein glycation [5]. Taken together, increased advanced glycation end-products (AGEs) have been related to the formation of atherosclerotic plaques and increased cardiovascular risk [13,14]. Lifeline cohort studies have shown that increased AGEs are independently related to BMI, age and HbA1c level [15]. Others have shown its increase in patients with visceral obesity, related to an increased prevalence of metabolic syndrome [16]. Similarly, our group, has previously published that the increase in AGE concentration in patients with severe obesity is clearly at the expense of those with metabolic syndrome, suggesting its determination as a way of identifying those patients with “metabolically diseased obesity” [17]. However, we are missing a study specifically designed to assess the impact of obesity, as measured by both BMI and body fat, on AGE levels. With this objective, and to verify if the accumulation of AGEs could help us to identify early and easily those people with a higher risk of metabolic syndrome, we have analyzed the population of the ILERVAS project. This large cohort included subjects with one or more cardiometabolic risk factors and different weight ranges.

## 2. Materials and Methods

### 2.1. Study Design

In this work, we analyze the information collected in the ILERVAS project (ClinTrials.gov Identifier: NCT03228459), a prospective study whose main goal was learning the prevalence of non-clinical atheromatous disease and occult kidney disease in a cohort with moderate cardiovascular risk [18,19]. Data were analyzed from 4254 people recruited between 2015 and 2018. Patients were recruited aged 45 to 70 years, with no previous cardiovascular event but at least one cardiometabolic risk factor (obesity, hypertension, dyslipidemia, smoking or first-degree relative with prematurity (<55 years in men, <65 in women) cardiovascular disease (myocardial infarction, stroke and peripheral arterial disease)). Those with diabetes, chronic kidney disease, active neoplasia, a life expectancy of less than 18 months and/or pregnancy were excluded.

The ILERVAS project protocol was approved by the ethics committee of the Arnau de Vilanova University Hospital (CEIC-1410) and written informed consent was acquired from all subjects. The ethical guidelines of the Declaration of Helsinki and Spanish legislation on the protection of personal data were also followed.

### 2.2. Definition of Cardiovascular Risk Factors

The diagnosis of dyslipidemia was obtained from patients who had an assigned code for disorders of lipoprotein metabolism and other lipidemias by means of the International Classification of Diseases (ICD-10) codes, namely E78.0–78.9 (pure hypercholesterolemia, pure hyperglyceridemia, mixed hyperlipidemia, hyperchylomicronemia, other hyperlipidemia, unspecified hyperlipidemia, lipoprotein deficiency, other disorders of lipoprotein metabolism and unspecified disorders of lipoprotein metabolism). A diagnosis of hypertension was obtained from patients coded for hypertensive diseases using ICD-10 codes, i.e., I10–I13 (essential hypertension, hypertensive heart disease, hypertensive renal disease and hypertensive heart and renal disease) and I15 (secondary hypertension).

Prediabetes was defined as a glycated hemoglobin (HbA1c) level between 39 to 47 mmol/mol (5.7 to 6.4%), and normal glucose metabolism as HbA1c <39 mmol/mol (<5.7%), agreeing with the American Diabetes Association guidelines. Smoking habits (never, former or current smoker) were also considered. Smokers who quit smoking a year or more before the visit were considered ex-smokers. As patients with diabetes were excluded from the ILERVAS project, this diagnosis was not considered a cardiovascular risk factor in our study.

The antihypertensive and lipid-lowering treatments that were prescribed in the ILERVAS population have been taken from the prescription and billing databases provided by CatSalut (Catalan Health Service), which were incorporated annually into the SIDIAP database. Antihypertensive medications include angiotensin-converting enzyme inhibitors, diuretics, type II aldosterone receptor antagonists, beta-blockers, calcium channel blockers and other antihypertensives. Lipid-lowering drugs included statins, fibrates, ezetimibe and omega-3 fatty acids.

### 2.3. Anthropometric Measures

Both weight and height were analyzed almost without clothing and without shoes with a precision of 0.5 kg and 1.0 cm, respectively [20]. Waist circumference was measured between the iliac crest and the lower rib in the horizontal plane with the subject standing and with a non-elastic tape to a precision of 0.1 cm [21]. To decrease interobserver and device variability, all anthropometric measures were performed by trained nurses under standardized conditions. The relative technical error of intra-rater measurement was less than 1% for height, weight and waist and circumferences.

BMI was obtained by weight (kg) divided by the square of body height (m), and obesity was classified according to clinical guidelines as BMI ≥30 kg/m^2^. The percentage of total body fat was estimated using the Body Adiposity Estimator of the Clínica Universidad de Navarra (CUN-BAE) using the formula: −44.988 + (0.503 × age) + (10.689 × sex) + (3.172 × BMI) − (0.026 × BMI^2^) + (0.181 × BMI) × sex) − (0.02 × BMI × age) − (0.005 × BMI^2^ × sex) + (0.00021 × BMI2 × age), where sex is 1 for women and 0 for men and age is in years [22].

For the estimation of central adiposity, in addition to waist circumference, the body roundness index was included. This index, suggested by Thomas et al., is based on a geometric model defined to quantify body circularity. Those with abdominal fat look like a perfect circle, compared to those with more linear figures. It was calculated as: WC (m)/(BMI^2/3^ × height (m))^1/2^ [23]. In addition, we evaluated the Hume index for the amount of lean mass based on the analysis of the body composition of the antipyrine dilution space through the formula: (0.29569 × weight) + (0.41813 × height) − 43.2933 [24].

### 2.4. Skin Autofluorescence

SAF was assessed using the AGE Reader™ device (DiagnOptics Technologies, Groningen, The Netherlands), a computerized non-invasive tool that quantifies AGE deposits in the forearm via the ultraviolet spectrum [25]. A device calibrated according to the manufacturer’s recommendations was used. Three analyses were carried out in areas free of tattoos, cosmetics or with a concentration of freckles or superficial vessels, and their mean value (arbitrary units: AU) was taken. Measurements made on the same day showed an overall Altman error rate of 5.03%, and intra-individual seasonal deviation showed an Altman error rate of 5.87% [25]. Since AGEs accumulate progressively with aging, there is a normal sum of AGEs at each age. When this number is higher than expected, the software classifies the patient as a “high AGE” individual. Therefore, participants in the ILERVAS project were classified as a group with “normal” and “high” SAF levels.

### 2.5. Statistical Methods

The Shapiro–Wilk test was used to estimate the normal distribution of the sample. Quantitative baseline characteristics were analyzed using the Mann–Whitney U test or Kruskal–Wallis test, and categorical characteristics using Pearson’s chi-squared test. Spearman’s correlation was used to assess the relationship between AGE levels and anthropometric data. Data are expressed as median and interquartile range or n (percentage). Patients were differentiated based on their elevated and normal SAF results. In addition, patients were also categorized according to the number of cardiovascular risk factors.

The evaluation of the diagnostic performance of the anthropometric formulas was carried out by analyzing the area under the receiver operating characteristic (ROC) curves and the Youden J statistic. The results of the area under the ROC curve were interpreted following the guidelines stipulated by the scientific community: excellent, between 0.9 and 1.0; good, between 0.8 and 0.9; fair, between 0.7 and 0.8; poor, between 0.6 and 0.7; and not useful, between 0.5 and 0.6. SSPS software (IBM SPSS Statistics for Windows, version 20.0., Armonk, NY, USA) was used for statistical analysis. Statistical significance was determined with a *p* value < 0.05.

## 3. Results

The main clinical and metabolic data according to the presence of SAF levels are shown in Table 1. The ILERVAS cohort consisted of 1115 (26.2%) individuals with elevated SAF values. This group of individuals were mainly smokers with a characteristic cardiovascular risk profile better than participants with normal SAF values. This high SAF group also received significant undertreatment with antihypertensive and lipid-lowering medications. However, no differences in the prevalence of obesity, according to BMI, were observed between groups (30.0 vs. 27.9%, *p* = 0.189). Similarly, no differences in total body fat percentage or estimated visceral adiposity and lean body mass were detected between the groups (Table 2).

**Figure 1:**
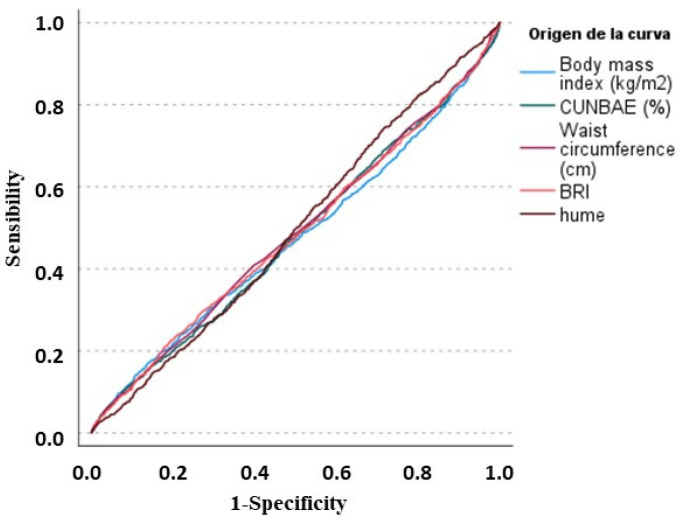
Receiver operating characteristic (ROC) curve analysis in the ILERVAS population to assess the diagnostic accuracy of obesity indices to identify patients with higher AGEs from those with normal AGEs.

Regarding the bivariate analysis, the SAF levels showed a very slight but positive correlation with the percentage of total body fat (estimated by the CUN-BAE formula) and abdominal adiposity (estimated by body roundness index) (Table 3). These correlations disappeared when anthropometric formulas such as BMI and waist circumference were used. In addition, a negative correlation with lean body mass was also observed. In the same way, the measures related to obesity and body composition had no power to identify the patients with higher levels of SAF, being in all cases areas under the ROC curve <0.52 (Figure 1).

**Figure 2:**
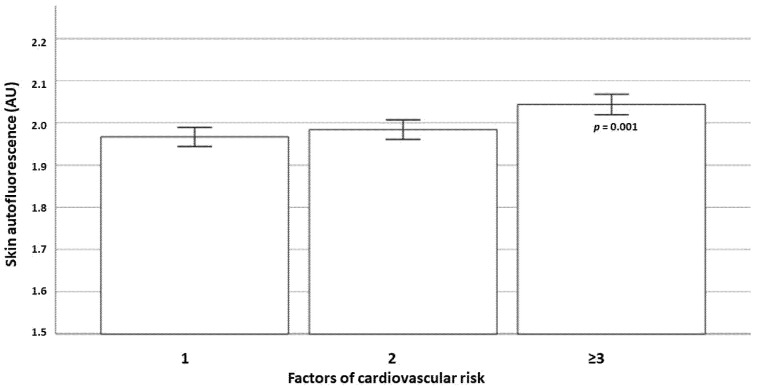
Results of the skin autofluorescence values in the entire population of the ILERVAS project according to the number of cardiovascular risk factors (a history of smoking habits, hypertension, dyslipidemia and prediabetes).

**Figure 3:**
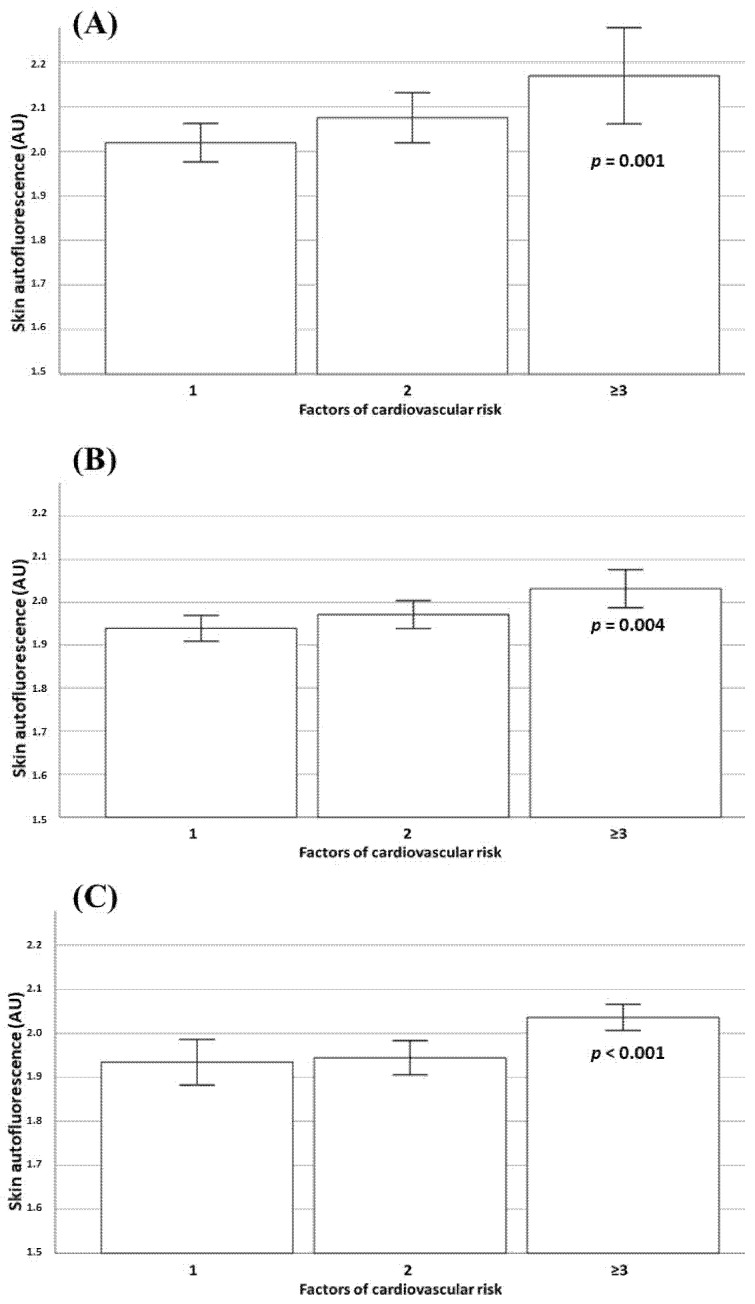
Results of the skin autofluorescence values in subjects with normal weight (**A**), overweight (**B**) and obesity (**C**) according to the number of cardiovascular risk factors (history of smoking habits hypertension, dyslipidemia and prediabetes).

We also analyzed SAF levels according to a number of cardiometabolic risk factors (dyslipidemia, hypertension, history of smoking and prediabetes) in both the total study population and according the degree of obesity. A progressive increase in SAF levels was observed in parallel with cardiovascular risk factors in the entire population (Figure 2). There were also significant differences in terms of skin autofluorescence values according to the number of cardiometabolic risk factors in subjects with normal weight, overweight and obesity (Figure 3).

Finally, the multivariable logistic regression model in patients with obesity according to their BMI showed that male sex, degree of obesity and the presence of three or more cardiovascular risk factors (prediabetes, smoking, hypertension and dyslipidemia) were independently associated with AGE levels (Table 4).

## 4. Discussion

In our middle-aged Caucasian population cohort, no significant increase in AGEs (measured as skin autofluorescence) was observed with respect to overall obesity or abdominal obesity. However, the subcutaneous deposition of AGE seems to be positively related to the prevalence of cardiometabolic risk factors, both in patients with and without obesity. Until now, when the relationship between obesity and AGE deposition has been evaluated, controversial data have been shown. For example, in a study of the child population, Lentferink et al. found a correlation of AGEs with the highest standard deviation of BMI, which disappeared when adjusting for skin type [26]. Likewise, in their study, Gogas et al. observed a positive correlation of AGEs with BMI, being higher in those with type 2 diabetes [27].

Visceral adipose tissue has been shown to have a greater inflammatory capacity, so it would be expected that this confers greater oxidative conditions favoring the formation of AGEs [28]. Despite this, Den Engelsen et al. did not observe significant differences in AGEs in those with or without central obesity measured by waist circumference. However, they demonstrated a progression: from those with healthy normal weight (1.63 ± 0.37 AU), increasing in those with abdominal obesity (1.74 ± 0.44 AU) and being even higher in subjects with abdominal obesity and comorbidities (1.87 ± 0.43 AU; *p* < 0.001) [16]. In the same study, after a medium follow-up period of 3 years after bariatric surgery, the SAF values did not change, although there was a marked reduction in weight and remission of comorbidities.

Elevated AGE levels have been linked to increased cardiometabolic risk, coronary artery disease and cardiovascular mortality [14,29]. Similarly, increased subcutaneous AGE content has previously been associated with increased atheromatous plaque burden in the ILERVAS project [13]. In the present study, we found differences when we assessed patients according to their cardiovascular risk, with SAF values that progressively increased according to the accumulation of cardiometabolic risk factors. Our results are in line with those of Koyama et al. who found a significant relationship between AGE receptors and metabolic syndrome, blood pressure, hypertriglyceridemia, and subclinical atheromatosis in both patients with and without diabetes [30]. Reinforcing the role of metabolic control in AGE deposition, plasma AGEs were higher in patients with type 2 diabetes and atherosclerotic disease than in patient with atherosclerotic disease without type 2 diabetes, especially in those with higher HbA1c levels in recent years (r = 0.46, *p* < 0.001) [31].

Other cardiovascular risk factors not evaluated in our study, such as chronic kidney disease or adherence to the Mediterranean diet, also cause an increase in AGE concentration [32]. In fact, studies based on dietary surveys have associated a low intake of exogenous AGEs with lower insulin resistance, TNF alpha levels, peripheral cell mononuclear cells and leptin concentration, as well as higher adiponectin, which ultimately means less proinflammatory activity [33]. Thus, adherence to the Mediterranean diet, an eating pattern associated with lower proinflammatory state, has been independently associated with AGEs, especially in those with a high consumption of vegetables, fruits and low sugar [34]. In our investigation it is also interesting to note that for the first time the negative but statistically significant link between the levels of SAF and lean body mass is shown.

Our research has some limitations. First, we do not use a precise measure of body composition to correlate with AGEs. However, anthropometric formulas have been validated with other gold standard tests such as dual-energy X-ray absorptiometry or magnetic resonance imaging. Second, we used an indirect test based on skin fluorescence to measure AGEs instead of a direct plasma test, but there is an extensive literature demonstrating the accuracy of this test compared to skin biopsy or plasma measurements. Third, an intrinsic characteristic of the ILERVAS study population is that participants have one or more cardiometabolic risk factors, so care must be taken when generalizing our results to the general population.

## 5. Conclusions

In conclusion, total obesity and visceral adiposity are not associated with a higher AGE deposit. The elevated levels of AGEs detected in subjects with obesity seem more related to the presence of cardiometabolic risk factors than to the percentage of body fat. With all this evidence, the measurement of SAF is a non-invasive test that can be helpful to identify those patients with unhealthy obesity, which opens the door to a new management of obesity in clinical practice.

## Figures and Tables

**Table 1:** Central clinical and metabolic data in the ILERVAS cohort according to skin autofluorescence values.

	Normal SAF (n = 3139)	High SAF (n = 1115)	*p*-Value
Women, n (%)	1576 (50.2)	548 (49.1)	0.543
Age (years)	57 (52–63)	57 (54–61)	0.003
Smoking habits (current and former), n (%)	1840 (58.6)	856 (76.8)	<0.001
Obesity diagnosis, n (%)	941 (30.0)	311 (27.9)	0.189
Blood hypertension diagnosis, n (%)	1296 (41.3)	390 (35.0)	<0.001
Antihypertensive drugs, n (%)	1036 (33.0)	313 (28.1)	0.002
Dyslipidemia diagnosis, n (%)	1635 (52.1)	495 (44.4)	<0.001
Lipid-lowering agents, n (%)	555 (17.7)	157 (14.1)	0.006
Prediabetes diagnosis, n (%)	1081 (34.4)	363 (32.6)	0.311

Data are expressed as a median (interquartile range) or n (percentage). Antihypertensive drugs include angiotensin-converting enzyme (ACE) inhibitors, diuretics, angiotensin-II receptor antagonists (ARA II), beta-blockers, calcium antagonists and other antihypertensives. Lipid-lowering treatments involve statins, fibrates, ezetimibe and omega-3 fatty acids.

**Table 2:** Data of the anthropometric indices in all individuals according to skin autofluorescence values.

	Normal SAF	High SAF	*p*-Value
Total adiposity			
BMI (kg/m^2^)	28.4 (25.7–31.6)	28.1 (24.7–31.8)	0.104
CUN-BAE (%)	35.9 (30.1–42.3)	35.3 (29.1–42.1)	0.132
Visceral adipose tissue			
Waist circumference (cm)	101 (94–108)	100 (94–108)	0.19
Body roundness index	5.68 (4.73–6.83)	5.58 (4.50–6.94)	0.127
Lean body mass			
Hume index (kg)	49.5 (43.0–56.2)	49.5 (43.4–55.5)	0.693

Data are expressed as a median (interquartile range). BMI: body mass index; CUN-BAE: Clínica Universidad de Navarra-Body Adiposity Estimator.

**Table 3:** Bivariate correlations of SAF with anthropometric formulas in the ILERVAS population.

	r	*p*
BMI (kg/m^2^)	−0.019	0.221
CUN-BAE (%)	0.080	<0.001
Waist circumference (cm)	0.005	0.746
Body roundness index	0.065	<0.001
Lean body mass (kg)	−0.122	<0.001

BMI: body mass index; CUN-BAE: Clínica Universidad de Navarra-Body Adiposity Estimator.

**Table 4:** The multivariable logistic regression model for high AGEs in subjects with obesity.

	Odds Ratio (95% CI)	*p*
Sex Women	Ref.	
Men	0.94 (0.79 to 1.11)	0.435
Age (years)	1.00 (0.99 to 1.01)	0.992
Body mass index (kg/m^2^)	1.03 (1.01 to 1.05)	0.006
Smoking status Never	Ref.	
Current or former	1.09 (0.092 to 1.28)	0.314
Cardiovascular risk factors 1	Ref.	
2	1.15 (0.98 to 1.36)	0.087
≥3	1.28 (1.04 to 1.58)	0.019
Hosmer–Lemeshow test of fit		0.492
Area under the ROC curve	0.74 (0.72 to 0.77)	<0.001

## Data Availability

The evidence presented in this investigation are accessible on request from the corresponding author. The data are not publicly presented due to the signed consent agreements around data sharing, which only allow access to the researchers of the ILERVAS project following the project purposes. Requestors wishing to access the data used in this work can make a demand to A.L. and M.B.-L. The request will be subjected to authorization and formal agreements regarding confidentiality and secure data storage being signed the data would be the provided.
